# Sequencing of 16S rRNA Gene: A Rapid Tool for Identification of *Bacillus anthracis*

**DOI:** 10.3201/eid0810.020391

**Published:** 2002-10

**Authors:** Claudio T. Sacchi, Anne M. Whitney, Leonard W. Mayer, Roger Morey, Arnold Steigerwalt, Ariana Boras, Robin S. Weyant, Tanja Popovic

**Affiliations:** * Centers for Disease Control and Prevention, Atlanta, Georgia, USA; † Adolfo Lutz Institute, São Paulo, Brazil; ‡ University Hospital for Infectious Diseases, Zagreb, Croatia

**Keywords:** Anthrax, *Bacillus anthracis*, Bioterrorism, 16S rRNA gene

## Abstract

In a bioterrorism event, a tool is needed to rapidly differentiate Bacillus anthracis from other closely related spore-forming Bacillus species. During the recent outbreak of bioterrorism-associated anthrax, we sequenced the 16S rRNA generom these species to evaluate the potential of 16S rRNA gene sequencing as a diagnostic tool. We found eight distinct 16S types among all 107 16S rRNA gene seqs fuences that differed from each other at 1 to 8 positions (0.06% to 0.5%). All 86 B. anthracis had an identical 16S gene sequence, designated type 6; 16S type 10 was seen in all B. thuringiensis strains; six other 16S types were found among the 10 B. cereus strains. This report describes the first demonstration of an exclusive association of a distinct 16S sequence with B. anthracis. Consequently, we were able to rapidly identify suspected isolates and to detect the B. anthracis 16S rRNA gene directly from culture-negative clinical specimens from seven patients with laboratory-confirmed anthrax.

The gram-positive, rod-shaped, and spore-forming bacterium Bacillus anthracis is the cause of the acute and often lethal disease anthrax. Phenotypic characteristics commonly used to differentiate B. anthracis from closely related B. cereus and B. thuringiensis, such as susceptibility to b-lactam antibiotics, lack of motility, lack of hemolysis on sheep blood agar (SBA) plate, and susceptibility to g-phage lysis, may vary among different Bacillus species strains, hampering their identification and differentiation. Phenotypically and genotypically B. thuringiensis can be differentiated from B. cereus by the presence of the CRY crystal protein and plasmid-encoded cry genes (1), but if this plasmid were lost, B. thuringiensis could no longer be distinguished from B. cereus (1). The sequence of the 16S rRNA gene has been widely used as a molecular clock to estimate relationships among bacteria (phylogeny), but more recently it has also become important as a means to identify an unknown bacterium to the genus or species level. The 16S rRNA gene sequences of B. anthracis, B. cereus, and B. thuringiensis have high levels of sequence similarity (>99%) that support their close relationships shown by DNA hybridization (2–7). A limited number of 16S rRNA sequences of B. anthracis (7 sequences), B. cereus (34 sequences), and B. thuringiensis (16 sequences) have been available at GenBank. Although those sequences are of different lengths and qualities, in complementary regions they differ from each other by no more than a few nucleotides. Therefore, this minimal level of diversity seen in the 16S rRNA of B. anthracis, B. cereus, and B. thuringiensis was thought to be an obstacle for using 16S rRNA gene sequencing to identify and differentiate these three species. The bioterrorism events of October 2001 prompted us to evaluate several new molecular approaches to rapidly identify B. anthracis. We determined the entire 16S rRNA sequences in a large number of representative strains of B. anthracis, B. cereus, and B. thuringiensis to evaluate the potential of 16S rRNA sequencing not only to rapidly identify B. anthracis in culture, but also to detect B. anthracis directly in clinical specimens.

## Materials and Methods

### Bacterial Strains

A total of 107 strains were included in this study. Of 86 B. anthracis isolates analyzed (Table 1), 18 were selected to represent a wide range of temporal (1937–1997), geographic (16 countries), and source diversity (soil, animals, or humans). Fourteen reference and standard strains, such as the Vollum, Ames, Pasteur, New Hampshire, V770, and Sterne strains, were also included. The remaining 54 strains were isolated from October to December 2001 during the bioterrorism-associated anthrax outbreak in the United States. Ten B. cereus and 11 B. thuringiensis strains were also analyzed by 16S rRNA sequencing. All strains were identified by standard microbiologic procedures and according to the Laboratory Response Network diagnostic criteria (9,10).

### Clinical Specimens

We analyzed 198 clinical specimens (76 blood, 30 tissue, 16 pleural fluid, 37 serum, 6 cerebrospinal fluid, and 33 other specimens). Sixty-nine specimens were obtained from patients with laboratory-confirmed anthrax (55 specimens from 11 inhalational cases and 14 from 7 cutaneous cases). DNA was extracted from fluid (200 µL) or small tissue specimens (<5 mm3) according to manufacturer's instructions with a Qiagen DNA Mini Kit (Qiagen, Valencia, CA). All 198 DNA samples were analyzed for 16S rRNA gene amplification and products sequenced.

### Polymerase Chain Reaction (PCR)

A 1,686-bp fragment of DNA, including the 1,554-bp 16S rRNA gene, was amplified from all 107 Bacillus species strains by using primers 67F and 1671R (Table 2). For clinical samples, we used the initial DNA amplicon as a template in a nested PCR with a second set of internal primers, 23F and 136R (Table 1). Both sets of primers were designed from the B. anthracis genome sequence (http://www.tigr.org). The full-length size of B. anthracis 16S rRNA gene (1,554 bp) was determined from an alignment of the 16S rRNA genes from Escherichia coli, Neisseria gonorrhoeae (GenBank accession nos. J01859 and X07714, respectively), and the 16S rRNA gene regions of the B. anthracis genome sequence (http://www.tigr.org). Whole cell suspensions or DNA extracts were used for PCR of isolates or clinical samples, respectively. For whole cell suspensions, a single colony from an SBA plate was resuspended in 200 µL of 10 mM Tris, pH 8.0. The suspension was put in a Millipore 0.22-µm filter unit (Millipore, Bedford, MA), heated at 95°C for 20 min, centrifuged at 8,000 rpm for 2 min, and then used for PCR. Each final PCR reaction (100 µL) contained 5 U of Expand DNA polymerase (Roche, Mannheim, Germany); 2 µL of whole cell suspension or DNA; 10 mM Tris-HCl (pH 8.0); 50 mM KCl; 1.5 mM MgCl2; 200 µM (each) dATP, dCTP, dGTP, and dTTP; and 0.4 µM of each primer. Reactions were first incubated for 5 min at 95°C. Then 35 cycles were performed as follows: 15 s at 94ºC, 15 s at the annealing temperature of 52ºC, and 1 min 30 s at 72ºC. Reactions were then incubated at 72ºC for another 5 min. The annealing temperature for the nested PCR was 50ºC. PCR products were purified with Qiaquick PCR purification kit (Qiagen).

### 16S rRNA Sequence Determination

The amplified products of approximately 1,686 bp (1,656 bp for nested PCR) were sequenced by using a modification of 16 primers as described (Table 2) (11). Sequencing was performed by using a Big Dye terminator cycle sequencing kit (Applied BioSystems, Foster City, CA). Sequencing products were purified by using Centri-Sep spin columns (Princeton Separations, Adelphia, NJ) and were resolved on an Applied BioSystems model 3100 automated DNA sequencing system (Applied BioSystems). The length of sequences obtained differed for each primer but were sufficient to provide 5- to 8-fold sequence coverage. An inner fragment of 1,554 bp was obtained and analyzed by using the GCG (Wisconsin) Package, v. 10.1, (Genetics Computer Group, Madison, WI). A number was assigned for each allele of 16S rRNA gene sequence in order of elucidation; a single base change or a mixed base (more than one nucleotide determined at a single position) was considered a new 16S type. When a novel 16S type, mixed base pairs, or any discrepancies in the alignment were obtained, the 16S rRNA gene amplification and sequencing of the entire gene or parts containing the problematic region were repeated.

### GenBank 16S rRNA Gene Sequences and Accession Numbers

Sixty 16S rRNA gene sequences of B. anthracis, B. cereus, and B. thuringiensis were available in GenBank. Thirty-nine of these sequences were incomplete, contained a large number of undetermined nucleotides, or were not associated with a specific strain identification, and therefore were not used in this study. The remaining 21 sequences were identified as eight B. anthracis (AF155950 [Ames]), (AF155951 [Delta Ames]), (AF176321 [Sterne]), (AF290552 [Sterne]), (AF290553 [Vollum]), (AF155950 [Ames]), (AF155951 [Delta Ames]), and (AF176321 [Sterne]); eight B. cereus (AF155952, AF155958, AF176322, AF290546, AF290547, AF290548, AF290550, and AF290555); three B. thuringiensis (AF155954, AF155955, and AF290549); and two B. mycoides (AF155956 and AF155957). A total of 114 16S rRNA gene sequences were determined in this study (107 from isolates [GenBank accession nos. in Table 1] and 7 from clinical specimens [GenBank accession nos. AY138359 to AY138365).

## Results

### 16S rRNA Gene Sequence Diversity

The 1,554-bp nucleotide sequences of the entire 16S rRNA gene from all 107 Bacillus species strains were aligned and compared. Differences were found at eight single nucleotide positions (positions 1, 2, 3, 4, 6, 9, 12, and 13), and no gaps were present. When 21 Bacillus 16S rRNA sequences from GenBank were added to the alignment, five additional positions with differences (positions 5, 7, 8, 10, and 11) were located (Table 3). The 13 positions of differences were distributed throughout the gene (Table 3). In six of these positions (positions 1, 2, 3, 4, 6, and 12), more than one nucleotide was detected (mixed nucleotides) (Table 3). These results indicated that the strain contained multiple rRNA operons with slightly different 16S rRNA gene sequences.

We found eight different 16S types among the 107 16S rRNA genes from our collection of isolates (Table 3). All 86 B. anthracis had an identical sequence, 16S type 6, containing a single mixed base, a W(A/T) at position 12, not found in the other two species. 16S type 10 was seen in all 11 B. thuringiensis strains, and a single mixed base pair was identified in all strains at position 6. Six other 16S types were found among the 10 B. cereus strains. Three additional 16S types were found among the 18 GenBank sequences that we analyzed. 16S types 1, 4, and 5 correlated to B. mycoides, B. thuringiensis, and B. cereus, respectively (Table 3). Five B. anthracis sequences from GenBank were identical to the 16S type 6 found in all our 86 B. anthracis isolates, and three were identical to the 16S type 7 found in B. cereus.

### 16S rRNA Sequencing Directly in Clinical Specimens

We detected 16S rRNA genes in 7 (3.5%) of 198 clinical samples: all were 16S type 6 characteristic for B. anthracis. None of the seven specimens were culture positive (Table 4), although all specimens had been collected from patients with laboratory-confirmed anthrax.

## Discussion

The goal of this study was to evaluate the potential of 16S rRNA sequencing to rapidly identify B. anthracis in cultures. We found that 16S rRNA genes of B. anthracis were highly conserved; only one 16S type (16S type 6) was identified in all 86 strains tested. However, not all B. anthracis 16S rRNA genes sequences in GenBank are type 6. Three of the eight B. anthracis 16S rRNA sequences are reported as type 7, a type that, in our study, we found exclusively among the B. cereus strains. The only difference between type 7 and type 6 is a mixed base pair at position 1146. The strain designations of two of these three 16S type 7 B. anthracis strains in GenBank are Ames and Sterne. We did not acquire these particular strains from the submitting laboratory, but the one Ames and two Sterne strains (obtained from different sources) in our collection were consistently type 6. A third Sterne strain 16S rRNA sequence in GenBank is also type 6.

One possible explanation for these different 16S rRNA sequencing results may be the use of different sequencing approaches, such as using cloned DNA versus genomic DNA as template. In sequencing clones, one allele may be missed if only a few clones are sequenced, not representing the total diversity. In this case, the position with the mixed base would not be detected. If both types 6 and 7 exist in B. anthracis, the difference may be due to recombination, mutation, or loss of an allele. The type 7 B. anthracis sequences in GenBank are unpublished; therefore, we do not know if the genes were cloned and, if so, how many clones were sequenced.

The complete B. anthracis genome was posted at http://www.tigr.org/tigr-scripts/ufmg/ReleaseDate.pl on May 7, 2002. The genome has 11 rRNA operons. There are 10 positions in the 16S rRNA gene where the nucleotides are not identical among the 11 rRNA operons, but the DNA sequencing software scores only one of them as a mixed base 100% of the time. This position is 1146, where five 16S rRNA genes contain Ts and six have As in a 54%:46% ratio. In this case, the base-calling software (GCG; Genetics Computer Group) always assigns a W at that position. At position 1137, there are seven Gs and four As, a 64%:36% ratio, but the position is scored as a G, the predominant base. In eight positions, a 9%:91% ratio is present. For example, at position 1047 are one T and 10 Cs. In these cases, the nucleotide is called as the predominant base by the base-calling software.

The quality of DNA sequences generated in laboratories has been greatly improved by the introduction of automated sequencing systems and DNA alignment software, but other factors, such as the purity of the DNA template and number of overlapping nucleotide fragments in the alignment, contribute to the reliability of the final sequence. Mixed base pairs are clearly the result of sequence differences between different rRNA operons and not due to any sequencing artifacts. In this study, the length of the fragment sequences varied for each primer, but they were of sufficient length to provide 5- to 8-fold sequence coverage in both directions. This 5–8 sequence overlap simplifies identifying and clarifying positions with double signals, increasing the confidence in our final consensus sequence. The occurrence of mixed base pairs in rRNA sequences is well known and accepted (15–19). The Ribosomal Database Project Web site shows that operon heterogeneity has been documented in several different bacterial species (http://rrndb.cme.msu.edu/rrndb/rrn_table.pdf). In addition, we did not observe mixed base pairs in single-copy genes such as pagA and a variety of others. A previous study of a small set of Bacillus strains isolated from soil demonstrated the diversity of 16S rRNA genes of both B. cereus and B. thuringiensis (15). Our results confirm the diversity among B. cereus strains, although we did not find diversity among B. thuringiensis strains. The lack of diversity in our collection of B. thuringiensis strains may be associated with natural selection with human host; 6 of 11 of our B. thuringiensis strains were isolated from humans.

### Direct Amplification of 16S rRNA from Clinical Samples

Even though B. anthracis is present at high levels (up to 108/mL) in the blood of patients with anthrax and will readily grow on standard bacteriologic media, as for other bacteria, specimens collected after the administration of antimicrobial therapy may fail to grow B. anthracis. Laboratory confirmation for the two patients with inhalational anthrax whose specimens were analyzed (patient #10i [12], and patient #11i [13]) was achieved by isolation and identification of B. anthracis from clinical samples at the medical facility where the patients were treated. Generally, for all patients, isolates themselves were forwarded to the appropriate public health laboratory and then to the Centers for Disease Control and Prevention for confirmatory identification and molecular subtyping, but the initial clinical specimens were not sent along with the isolates. With few exceptions, clinical specimens available for analysis from these two patients and from other patients with inhalational anthrax were collected after initiation of antimicrobial therapy, resulting in few culture-positive results. For 3 of the 11 inhalational patients, laboratory confirmation was based on two of three available supportive tests, including PCR targeting two plasmid and one chromosomal target (14), immunohistochemistry or a reactive anti-protective antigen title (immunoglobulin G ELISA) (12,20). Laboratory confirmation for the two cutaneous cases with skin biopsies analyzed in this study was indeed achieved by these supportive laboratory tests: one case was confirmed by immunohistochemistry and a reactive anti-protective antigen titer (IgG ELISA). For the other case all three supportive laboratory tests were positive.

Previously, strains having <3% difference between their 16S rRNA genes were considered the same species (21). However, differences between 16S rRNA genes for some Bacillus species, such as B. anthracis, B. cereus, and B. thuringiensis, are <1% (1) . Such small differences (e.g., one base between sequences or partial matches at a single nucleotide position in the 16S rRNA gene) have not been used for species differentiation. Our study clearly demonstrates that such small differences might be important for species identification. DNA-DNA hybridization and 16S rRNA sequencing studies have shown that these three Bacillus species are closely related and probably represent a single species (3,6,7). If the three were classified as a single species, 16S rRNA sequencing appears to have the potential to differentiate strains at the subspecies level.

Although pXO1 and pXO2 plasmids must be detected to confirm the virulence of B. anthracis, 16S rRNA sequencing has a powerful capacity to rapidly identify B. anthracis and other species. Although further studies are needed to fully evaluate 16S sequencing as a diagnostic assay, its value as a tool for rapid initial screening in outbreak investigations has been demonstrated.

**Table 1. **Descriptions and GenBank accession numbers of 107 *Bacillus *species strains analyzed in this study

**Table:** 

Species		No.	Identification	GenBank 16S rRna gene accession number	Geographic and/or temporal origin^a^	16S rRNA type	MLVA genotype^b^
*B. anthracis*	Diversity	18	2000031650	AY138379	Human, Turkey, 1991	6	23
	collection		2000031651	AY138372	Bovine, France, 1997	6	80
			2000031652	AY138374	Human, US, 1952	6	68
			2000031653	AY138373	Wool, Pakistan, 1976	6	69
			2000031655	AY138376	Cow, China	6	57
			2000031656	AY138375	Ames	6	62
			2000031657	AY138382	Bovine	6	10
			2000031659	AY138377	Human, Turkey, 1984	6	28
			2000031660	AY138378	Bovine, US, 1937	6	25
			2000031661	AY138369	Human, South Korea, 1994	6	34
			2000031662	AY139368	Zebra, Namibia	6	35
			2000031663	AY138381	Bovine, Poland	6	15
			2000031664	AY138383	Porcine, German, 1971	6	38
			2000031665	AY138366	Bovine, Argentina	6	45
			2000031666	AY138371	UK	6	77
			2000031667	AY138380	Sheep, Italy, 1994	6	20
			2000031670	AY138367	Human, Turkey, 1985	6	41
			2000031671	AY138370	Bovine, Zambia	6	30
	Standard and	14	Ames	AY138358	Ames	6	62
	reference		2002007651	AY138355	Sterne, Chile	6	ND
	strains		2002007650	AY138356	Sterne, Chile	6	ND
			2002007649	AY138357	Pasteur, Chile	6	ND
			2000031887B	AY138347	Vaccine	6	ND
			2000031666	AY138352	Vollum	6	77
			2000031368	AY138350	Vollum	6	77
			2000031244	AY138354	Vollum	6	*
			2000031078	AY138351	Vollum M36	6	ND
			2000031076	AY138353	Vollum	6	*
			2000031259	AY138346	Pasteur	6	**
			2000031075	AY138345	Sterne	6	*
			2000031887	AY138348	V770-NP1-R	6	*
			2000031136	AY138349	New Hampshire	6	73
	Outbreak strains	54		AY138291 to AY138344	US Oct/Dec 2001	6	62
*B. cereus*		10	2000031486	AY138272	Human, US, 1994	12	NA
			2000031491	AY138276	Human, US, 1997	7	NA
			2000031498	AY138274	Human, US, 1979	9	NA
			2000031503	AY138277	Human, US, 1999	7	NA
			2000031513	AY138279	Human, US, 1986	13	NA
			G3317	AY138278	Human, Israel, 1989	7	NA
			G8639	AY138271	Milk, Bolivia, 1993	3	NA
			G9667	AY138273	Human, US, 1995	12	NA
			H1439	AY138270	Human, US, 2000	2	NA
			ATCC 14579	AY138275	1887	9	NA
*B. thuringiensis*		11	2000031482	AY138290	Human, US, 1989	10	NA
			2000031485	AY138289	Spray, US, 1993	10	NA
			2000031494	AY138288	Human, US, 1985	10	NA
			2000031496	AY138287	Human, US, 1981	10	NA
			2000031508	AY138286	Human, US, 1985	10	NA
			2000031509	AY138285	Human, US, 1985	10	NA
			2002007400	AY138283	Powder, US, 2001	10	NA
			2002017401	AY138284	Powder, US, 2001	10	NA
			2000032755	AY138282	Environment, US, 2000	10	NA
			2000032757	AY138280	Environment, US, 2000	10	NA
			2000032756	AY138281	Human, US, 1981	10	NA
^a^Date and source of isolation are provided when available; *, lacking pXO2; **, lacking pXO1. ^bN^D, MLVA genotype not done (8). NA, not applicable.							

 **Table 2.** Primers used for amplification and sequencing of the 16S rRNA gene of *Bacillus anthracis*, *B. thuringiensis*, and *B. cereus*^a^

**Table:** 

**Generic primers used for 16S rRNA amplification**	
8F	5´AGT TGA TCC TGG CTC AG 3´
1492R	5´ACC TTG TTA CGA CTT3´
**Primers for amplification of the 16S rRNA gene**	
67F	5´TGA AAA CTG AAC GAA ACA AAC 3´
1671R	5´CTC TCA AAA CTG AAC AAA ACG AAA 3´
**Inner primers used for nested PCR on clinical samples**	
23F	5´ACA AAC AAC GTG AAA CGT CAA 3´
136R	5´AAA CGA AAC ACG GAA ACT T 3´
**Primers used for sequencing of the 16S rRNA gene**	
104F	5´GGA CGG GTG AGT AAC ACG TG 3´
104R	5´CAC GTG TTA CTC ACC CGT CC 3´
1230F	5´TAC ACA CGT GCT ACA ATG 3´
1390F	5´GGG CCT TGT ACA CAC CG 3´
1390R	5´CGG TGT GTA CAA GGC CC 3´
8F	5´AGT TGA TCC TGG CTC AG 3´
357F	5´TAC GGG AGG CAG CAG 3´
357R	5´CTG CTG CCT CCC GTA 3´
530F	5´CAG CAG CCG CGG TAA TAC 3´
530R	5´GTA TTA CCG CGG CTG CTG 3´
790F	5´ATT AGA TAC CCT GGT AG 3´
790R	5´CTA CCA GGG TAT CTA AT 3´
981F	5´CCC GCA ACG AGC GCA ACC C 3´
981R	5´GGG TTG CGC TCG TTG CGG G 3´
^a^Primers 67F and 1671R or primers 23F and 136R were also used for 16S sequence on isolates or clinical samples, respectively.	

 **Table 3. **16S rRNA gene types identified among 125 *Bacillus* spp. strains analyzed in this study (n=107) and available at GenBank (n=18)

**Table:** 

			Positions^a^												
16S type	*Bacillus *species	No. of strains	1 (77)	2 (90)	3 (92)	4 (182)	5 (189)	6 (192)	7 (200)	8 (208)	9 (1,015)	10 (1,036)	11 (1,045)	12 (1,146)	13 (1,462)
**16S types identified in 107 strains in this study**															
2	*cereus*	1	R^b^	Y	W	C^c^	A	T	T	G	A	T	A	A	A
3	*cereus*	1	G	C	A	Y	A	T	T	G	A	T	A	A	A
6	*anthracis*	86	A	T	T	C	A	C	T	G	C	T	A	W	T
7	*cereus*	3	A	T	T	C	A	C	T	G	C	T	A	A	T
9	*cereus*	2	A	T	T	C	A	C	T	G	A	T	A	A	T
10	*thuringiensis*	11	A	T	T	C	A	Y	T	G	A	T	A	A	T
12	*cereus*	2	A	T	T	Y	A	T	T	G	A	T	A	A	T
13	*cereus*	1	A	T	T	C	A	C	T	G	C	T	A	T	T
**16S types identified in strains available at GenBank^d^**															
1	*mycoides*	2	A	T	T	C	C	C	G	C	C	C	G	A	^e^
4	*thuringiensis*	3	G	C	A	C	A	T	T	G	A	T	A	A	^e^
5	*cereus*	8	G	C	A	C	A	C	T	G	A	T	A	A	^e^
7	*anthracis*	3	A	T	T	C	A	C	T	G	C	T	A	A	T
^a^Numbers refer to the number of positions where mismatches are found. Numbers in parentheses refer to positions in the 16S rRNA gene. ^b^R refers to a purine (A or G) at that position; Y refers to a pyrimidine (C or T) at that position; and W refers to an A or T at that position. ^c^A, C, G, and T refer to the four deoxynucleotides that DNA comprises. ^d^Five additional positions of differences (positions 5, 7, 8, 10, and 11) were found when GenBank sequences were used. ^e^The last position (position 13) on 16S types 1, 4, and 5 is missing because those GenBank sequences are shorter.															

 **Table 4. **Results of laboratory testing on seven clinical samples in which 16S type 6 was identified

**Table:** 

Patient			Clinical specimens		
ID^a^	Diagnosis	Laboratory confirmation^b^	Type	Culture	*B. anthracis*-specific PCR^b^
2i	Inhalational anthrax	IHC + PCR of pleural fluid; serology	Tissue	Neg	Neg
10i	Inhalational anthrax	*B. anthracis* isolated from blood and pleural fluid	Pleural fluid	Neg	Pos
			Pleural fluid	Neg	Pos
			Blood	Neg	ND
			Lymph node	Neg	Pos
11i	Inhalational anthrax	*B. anthracis *isolated from blood	Lymph node	Neg	Pos
7c	Cutaneous anthrax	IHC + PCR on skin biopsy	Skin from forehead	Neg	Pos
^a^Patient identification numbers are described in references 12 and 13; I, inhalational case; C, cutaneous case; PCR, polymerase chain reaction. ^b^The immunohistochemical (IHC), serologic, and PCR results are described in reference 14.					
